# Effects of Probiotic Supplementation on Dyslipidemia in Type 2 Diabetes Mellitus: A Meta-Analysis of Randomized Controlled Trials

**DOI:** 10.3390/foods9111540

**Published:** 2020-10-26

**Authors:** Chen Wang, Chengcheng Zhang, Sijia Li, Leilei Yu, Fengwei Tian, Jianxin Zhao, Hao Zhang, Wei Chen, Qixiao Zhai

**Affiliations:** 1 State Key Laboratory of Food Science and Technology, Jiangnan University, Wuxi 214122, China; 7180112085@stu.jiangnan.edu.cn (C.W.); 7160112075@vip.jiangnan.edu.cn (C.Z.); 6180112032@stu.jiangnan.edu.cn (S.L.); leileiyu@jiangnan.edu.cn (L.Y.); fwtian@jiangnan.edu.cn (F.T.); jxzhao@jiangnan.edu.cn (J.Z.); zhanghao@jiangnan.edu.cn (H.Z.); chenwei66@jiangnan.edu.cn (W.C.); 2 School of Food Science and Technology, Jiangnan University, Wuxi 214122, China; 3 National Engineering Research Center for Functional Food, Jiangnan University, Wuxi 214122, China; 4 Wuxi Translational Medicine Research Center and Jiangsu Translational Medicine, Research Institute Wuxi Branch, Wuxi 214122, China; 5 (Yangzhou) Institute of Food Biotechnology, Jiangnan University, Yangzhou 225004, China; 6 Beijing Innovation Centre of Food Nutrition and Human Health, Beijing Technology and Business University (BTBU), Beijing 100048, China; 7 International Joint Research Laboratory for Probiotics at Jiangnan University, Wuxi 214122, China

**Keywords:** type 2 diabetes mellitus (TDM), dyslipidemia, meta-analysis, probiotic, intervention, multispecies probiotics

## Abstract

The effectiveness of probiotic consumption in controlling dyslipidemia in type 2 diabetes mellitus (T2DM) has been unclear. We reviewed relevant randomized controlled trials (RCTs) to clarify the effect of probiotic intake on dyslipidemia in T2DM patients. The Web of Science, Scopus, PubMed and Cochrane Library databases were used for searching relevant RCTs published up to October 2020. The total cholesterol (TC), triglyceride (TG), low-density lipoprotein cholesterol (LDL-C) and high-density lipoprotein cholesterol (HDL-C) concentrations were selected as the primary indicators for dyslipidemia. The results of 13 eligible RCTs showed that probiotic intake could significantly reduce TC (SMD: −0.23, 95% CI: (−0.37, −0.10)) and TG (SMD: −0.27, 95% CI: (−0.44, −0.11)) levels, but did not regulate LDL-C or HDL-C concentrations. Subgroup analysis showed that multispecies probiotics (≥two species), but not single-species probiotics, significantly decreased TC and TG concentrations. Furthermore, powder, but not liquid, probiotics could reduce TC and TG concentrations. This meta-analysis demonstrated that probiotic supplementation is helpful in reducing TC and TG concentrations in T2DM patients. However, more well-controlled trials are needed to clarify the benefits of probiotics on dyslipidemia in T2DM patients.

## 1. Introduction

Type 2 diabetes mellitus (T2DM) is a complex metabolic disorder characterized by islet beta cell failure and insulin resistance [1]. It is the most prevalent type of diabetes patients [2]. Based on previous studies, T2DM is considered as a multifactorial disease, with genetic predisposition, environmental factors and behavioral changes contributing to disease incidence [3,4]. T2DM can lead to a series of severe complications, which include cardiovascular disease, neuropathy and nephropathy [5], making it a serious threat to human health. Recently, it has been considered as a major global public health concern due to the increasing number of affected patients and the reducing age of disease onset [6].

Previously, some studies have shown that more than 50% of T2DM patients present with dyslipidemia [7,8]. Dyslipidemia is characterized by an increase in total cholesterol (TC), low-density lipoprotein cholesterol (LDL-C) and triglyceride (TG) concentrations, and a decrease in high-density lipoprotein cholesterol (HDL-C) concentrations, either occurring individually or in various combinations [9]. Some previous studies have confirmed the relationship between dyslipidemia and T2DM [10,11]. They have shown that dyslipidemia is highly prevalent (>75%) in T2DM patients [12], which manifested as the elevated plasma concentrations of LDL-C particles, and low concentrations of HDL-C in T2DM patients [13,14]. In another study, 108 adult T2DM patients were recruited by the Nnamdi Azikiwe University Teaching Hospital Nnewi to evaluate the potential pattern of dyslipidemia among T2DM patients [7]. The results showed that 24.1% of the patients had single dyslipidemia and 66.6% had combined dyslipidemia [7]. Therefore, the influence of dyslipidemia on the development and progression of T2DM should not be overlooked. Given the close relationship between T2DM and dyslipidemia, ongoing research has focused on searching for effective methods to relieve T2DM.

The results of clinical trials indicate that probiotics may have potential therapeutic effects on T2DM. It was found that probiotic consumption could be helpful in alleviating the dysregulation of blood lipids and blood pressure [15], and decreasing cholesterolemia [16]. After that, several meta-analyses have attempted to elucidate the effects of probiotics on T2DM. One study [17] found that probiotics intake could markedly increase HDL-C concentrations, but had no significant effects on LDL-C, TC or TG levels compared with the control groups. In contrast, another meta-analysis [18], which included 11 eligible RCTs, suggested that probiotic consumption remarkably decreased TC, TG and LDL-C concentrations in the T2DM groups, compared with the placebo groups. The variable results reported by these meta-analyses have led to controversy. These variable results may be attributable to the variabilities in eligibility criteria, study selection, number of studies included and targeted outcomes.

Thus, a meta-analysis with a larger sample size and more recent reports is necessary to clarify the potential effects of probiotic intake on dyslipidemia in T2DM patients. We aim to analyze all recent (up to February 2020) eligible RCTs and determine the clinical benefits of probiotic intake in T2DM patients, including changes in TC, TG, LDL-C and HDL-C concentrations. Subgroup analyses were performed to clarify the effects of numbers of probiotic species, body mass index (BMI), intervention type and supplementation duration on controlling dyslipidemia in T2DM patients. Notably, our meta-analysis differs from previous studies, particularly with respect to the major clinical endpoints and the participant characteristics. Our analysis included more RCTs (*n* = 15, 884 participants) than previous studies. Additionally, two recent clinical studies by Sabico et al. [19] and Razmpoosh et al. [20] that were not analyzed in previous meta-analyses were also included in our study.

## 2. Materials and Methods

### 2.1. Search Strategy and Eligibility Criteria

This study was completed according to the guidelines of Cochrane [21] and PRISMA [22], respectively. The PubMed (up to October 2020), Scopus (up to October 2020) Web of Science (1950 to October 2020) and Cochrane Library (up to October 2020) databases were used for searching relevant RCTs. Besides this, the following terms were used to identify eligible studies: (Random* OR blind* OR allocate* OR assign* OR trial* OR crossover* OR cross-over) AND (T2DM* OR T2D* OR type 2 diabetes mellitus* OR type 2 diabetes) AND (probiotics* OR probiotic* OR Bifidobacterium* OR Lactobacillus* OR Streptococcus* OR Saccharomyces* OR Bacillus*).

This step was completed independently by two authors (Chen Wang and Chengcheng Zhang). The inclusion criteria for eligible RCTs were as follows: (1) The participants were T2DM patients; (2) the participants treated with probiotics formed the treatment group, and a placebo was used as the control treatment; (3) the RCT measured the outcomes for at least one of the set parameters (TC, TG, LDL-C and HDL-C concentrations); and (4) the RCT had a parallel or crossover design but did not include reviews, protocols, letters or case reports.

### 2.2. Data Items and Data Collection Process

Two authors (Chengcheng Zhang and Sijia Li) extracted the following data from the included RCTs: the first author’s name, trial registration number, publication year of included RCT, study design, participants’ basic characteristics (including number, sex, mean age and BMI), country, sample size, number of probiotic species, type of intervention, dosage and duration of probiotic treatment and the primary outcomes (including TC, TG, LDL-C and HDL-C concentrations).

### 2.3. Assessment of Risk of Bias

The Cochrane risk-of-bias tool [23] was used to detect the risk of bias. The assessment was performed by two authors independently (Chen Wang and Jianxin Zhao), and a third author’s opinion (Wei Chen) was sought to resolve any disagreement.

### 2.4. Data Synthesis and Analysis

Five steps were completed by two authors (Chen Wang and Qixiao Zhai) to synthesize and analyze the data. First, all of the data were included and analyzed using the Review Manager version 5.3 software (Cochrane Collaboration, Oxford, England). The Cochran’s Q-test [24] and reported I-square (I^2^) [25] were used for assessing the heterogeneity of the effect of probiotic intake. The I^2^ value of heterogeneity (I^2^) was classified as per previous studies [18,26]: <25% (represents low heterogeneity), 25–75% (represents moderate heterogeneity), and >75% (represents high heterogeneity). Standardized mean differences (SMDs) and their 95% confidence intervals (CIs) were used for calculating the pooled data. Two methods were used for assessing the possibility of high heterogeneity, as follows: (1) Subgroup analyses (including BMI, number of species, types of interventions and duration of probiotic); (2) sensitivity analysis (to investigate which studies caused the high heterogeneity and how studies contributed to the meta-analysis) [27]. The Begg’s and Egger’s tests (with *p* < 0.05 regarded as significant) and visual inspection of funnel plots [28] were used to detect possible publication bias in individual analyses. However, this study has never been registered in the international prospective register of systematic reviews (PROSPERO).

## 3. Results

### 3.1. Characteristics of Included Papers

**Figure 1:**
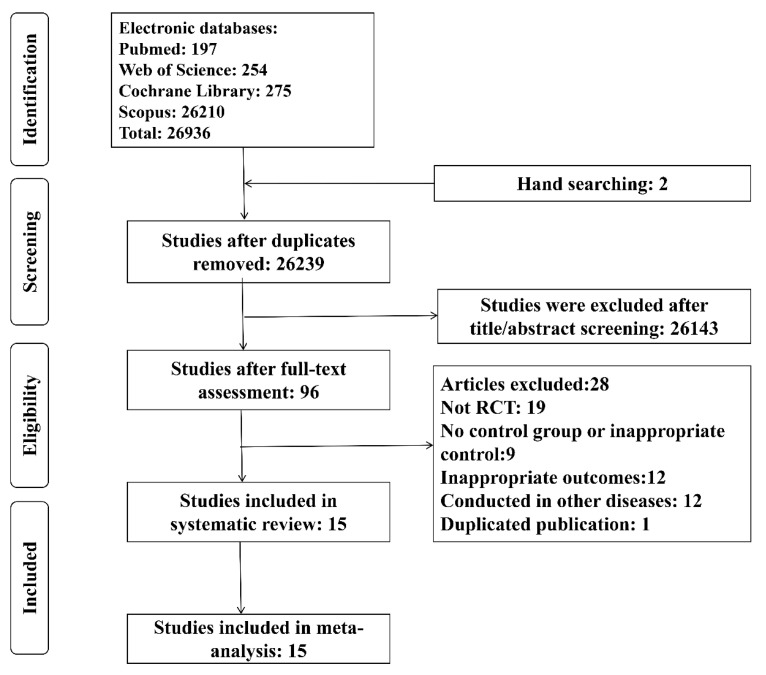
Flow diagram of the present meta-analysis.

As shown in Figure 1, our initial search yielded 26,936 articles, 697 of which were deleted following the removal of duplicates. Of the remaining studies, 15 RCTs were selected for inclusion in the present study after screening. These studies were conducted in Iran [20,29,30,31,32,33,34,35,36], Malaysia [37], China [38], Sweden [39], Saudi Arabia [19], Japan [40] and Brazil [41]. The included studies comprised two double-blind randomized cross-over controlled clinical trials [30,31], eight double-blind randomized controlled clinical trials [19,20,29,32,33,36,38,39], three randomized, double-blind, parallel-group, placebo-controlled trials [34,37,41], one single-blinded clinical trial [35], and one interventional RCT [40]. Probiotics were supplemented in different forms, which included fermented milk [34,36,40,41], yogurt [33], freeze-dried powder in a capsule [19,20,29,32,35,38,39] or sachet [37], or symbiotic food [30,31]. The studies administered either one [30,31,34,38,39,40], two [33,41], three [36], four [35], six [37] or more [19,20,29,32] bacterial species. The characteristics of these included RCTs have been listed in Table 1.

### 3.2. Risk of Bias in Individual Studies

**Figure 2:**
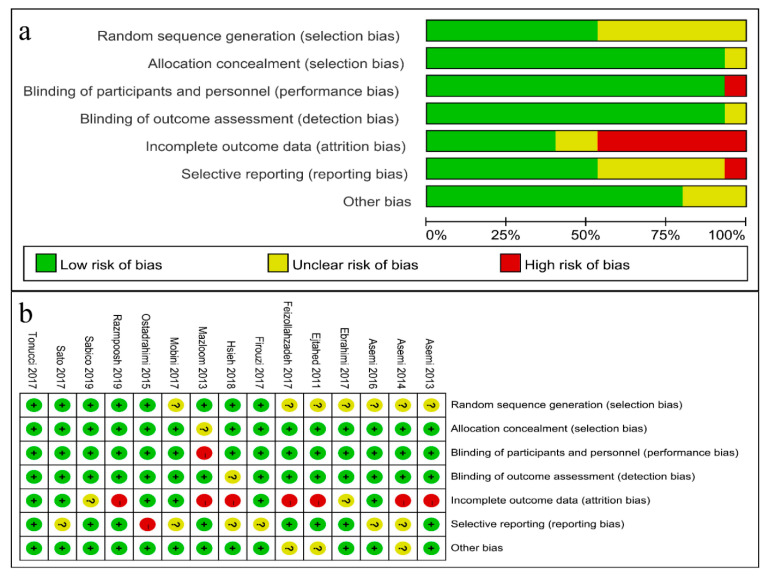
Risk-of-bias assessment for the included RCTs. Risk of bias summary (**a**) and graph (**b**). Note: “+”: low risk, “?”: unclear risk, “−”: high risk.

As shown in Figure 2, selective reporting bias was present in seven studies [29,30,31,32,33,34,39], and one study did not mention the method of randomization [35]. Attrition bias can be found in nine RCTs due to the withdrawal of several participants during the study period [19,20,29,30,32,33,34,35,38]. Overall, six RCTs were defined as methodologically sound and of high quality [19,20,36,37,40,41]. The other nine trials were rated as being of fair quality for having unclear risk of bias, particularly with respect to selection bias, attrition bias and reporting bias. In total 12, RCTs were registered as clinical trials, whereas three studies were not registered anywhere [29,31,35] (Table 1). Besides this, the Systematic Reviews and Meta-Analyses (PRISMA) statement is shown in Supplementary Table S1.

### 3.3. Effects of Probiotics on Blood Lipid Profiles

#### 3.3.1. Plasma TC Levels

**Figure 3:**
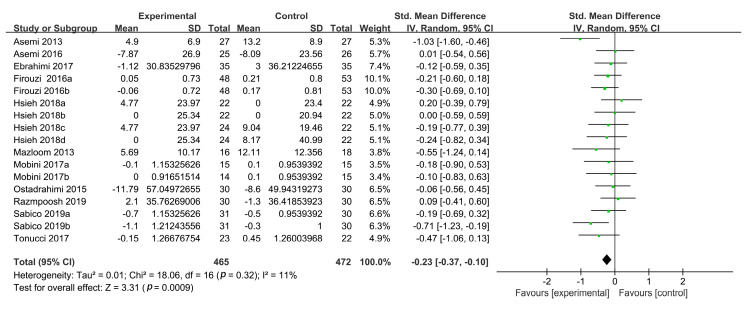
Forest plot of the effects of probiotics on TC concentrations.

In total, 13 studies reported plasma TC concentrations, 3 of which had two intervention groups [19,37,39] and 1 had four intervention groups [38]. All experimental groups in these studies were defined as separate studies for inclusion in this study (*n* = 19). Compared with the control treatment, probiotic supplementation did not reduce TC concentrations (SMD: −0.19, 95% CI: (−0.48, 0.10), *p* = 0.20) in the random-effects model, and the heterogeneity was high (*n* = 19; I^2^ = 82%). The results of sensitivity analysis showed that the high heterogeneity was derived from two studies in particular [38]. The exclusion of these two RCTs reduced the heterogeneity to 11%, revealing a reduction in the TC concentrations (SMD: −0.23, 95% CI (−0.37, −0.10), *p* = 0.0009) (Figure 3).

#### 3.3.2. Plasma TG Levels

**Figure 4:**
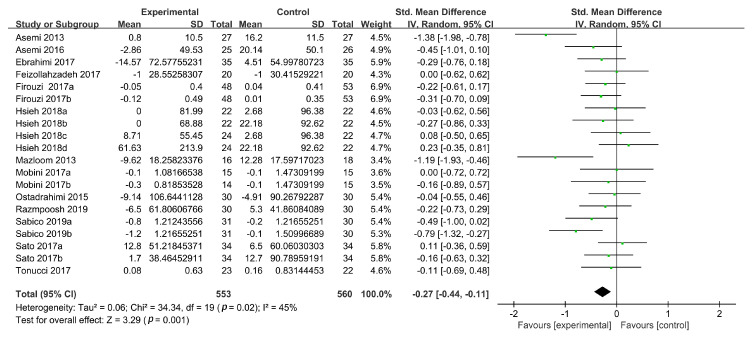
Forest plot of the effects of probiotics on TG concentrations.

TG concentrations were reported in 15 studies, 4 of which had two intervention groups [19,37,39,40] and 1 had four intervention groups [38]. In total, 22 intervention groups were evaluated for TG concentrations in this meta-analysis. Probiotic intake did not reduce TG concentrations (SMD: −0.19, 95% CI: (−0.44, 0.06), *p* = 0.15) in T2DM patients in the random-effects model. In addition, significant heterogeneity (*n* = 22; I^2^ = 79%) was found between the studies. A sensitivity analysis showed that the high heterogeneity was derived from two studies [30,33]. Upon the exclusion of these two studies, the heterogeneity was reduced to an acceptable level (I^2^ = 45%), and a significant reduction in the TG concentrations could be observed (*n* = 20; SMD: −0.27, 95% CI: (−0.44, −0.11), *p* = 0.001) (Figure 4).

#### 3.3.3. Plasma LDL-C Levels

**Figure 5:**
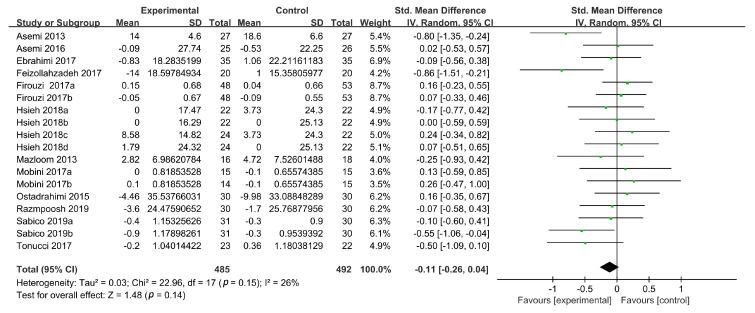
Forest plot of the effects of probiotics on LDL-C concentrations.

LDL-C concentrations were reported in 14 studies, 3 of which had two intervention groups [19,37,39] and 1 had four intervention groups. In total, 20 intervention groups from the included RCTs were evaluated. The SMD was calculated to evaluate the role of probiotic supplementation in managing the LDL-C concentration. Compared with the placebo treatment, probiotic consumption did not reduce LDL-C concentrations (*n* = 20; SMD: −0.14, 95% CI: (−0.39, 0.11), *p* = 0.26), with a high heterogeneity (I^2^ = 76%). A sensitivity analysis showed that the high heterogeneity was derived from two studies [30,33]. The omission of these two RCTs reduced the heterogeneity to an acceptable level (I^2^ = 26%), but the reduction in the LDL-C concentrations as a result of probiotic consumption compared with the placebo treatment was still not significant (SMD: −0.11, 95% CI: (−0.26, 0.04), *p* = 0.14) (Figure 5).

#### 3.3.4. Plasma HDL-C Levels

**Figure 6:**
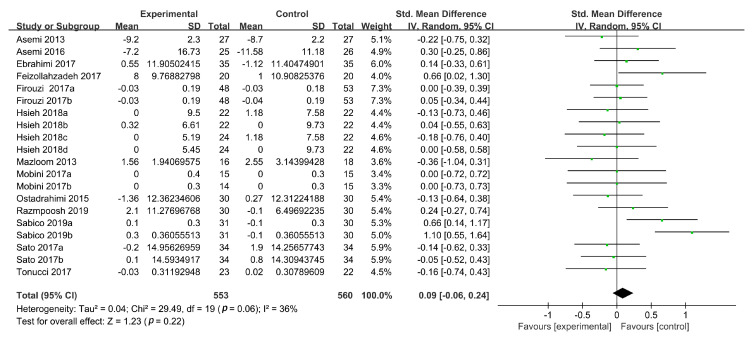
Forest plot of the effects of probiotics on HDL-C concentrations.

HDL-C concentrations were reported in 15 studies, 4 of which contained two intervention groups [19,37,39,40] and 1 included four intervention groups [38]. These groups were divided and treated as separate RCTs, yielding 22 studies for the evaluation of HDL-C concentrations. The calculated SMDs showed that probiotic intervention significantly increased the HDL-C levels (SMD: 0.25, 95% CI: (0.01, 0.50)), but heterogeneity was high between the studies (*n* = 22; I^2^ = 78%; *p* = 0.05). A sensitivity analysis identified two studies [30,33] that contributed to the high heterogeneity. After excluding these RCTs, the heterogeneity reduced to 36%, but the increase in the HDL-C concentrations caused by probiotic consumption (SMD, 0.09, 95% CI: (−0.06, 0.24), *p* = 0.22) became nonsignificant compared with the placebo group (Figure 6).

### 3.4. Subgroup Analysis

As shown in Table 2, the subgroup analysis based on the number of probiotic species revealed that multispecies probiotics, but not single-species probiotics, could significantly decrease the TC (SMD: −0.32, 95% CI: (−0.52, −0.12); *p* = 0.001; I^2^ = 34%) and TG (SMD: −0.46, 95% CI: (−0.71, −0.21); *p* = 0.0003; I^2^ = 58%) concentrations. A long duration (≥8 weeks) of probiotic consumption was helpful in reducing TC concentrations (SMD: −0.25, 95% CI: (−0.43, −0.07); *p* = 0.006; I^2^ = 23%). The effects of the duration of probiotic consumption (≥8 weeks or <8 weeks) showed a similar trend for TG concentrations. Compared to patients with low BMI (<29 kg/m^2^), those with high BMI (≥29 kg/m^2^) showed reduced TC (SMD: −0.34, 95% CI: (−0.57, −0.11); *p* = 0.003; I^2^ = 33%) and TG (SMD: −0.48, 95% CI: (−0.75, −0.20); *p* = 0.0006; I^2^ = 53%) concentrations. Further, the subgroup analysis based on the type of intervention revealed that probiotic intake in powder form, but not liquid form, could reduce the TC (SMD: −0.23, 95% CI: (−0.39, −0.08); *p* = 0.003; I^2^% = 18) and TG (SMD: −0.36, 95% CI: (−0.56, −0.15); *p* = 0.0005; I^2^% = 51) concentrations. Notably, the subgroup analyses based on the number of probiotic species, intervention type, BMI and duration showed no significant role of probiotics in regulating LDL-C and HDL-C concentrations.

### 3.5. Publication Bias

**Figure 7:**
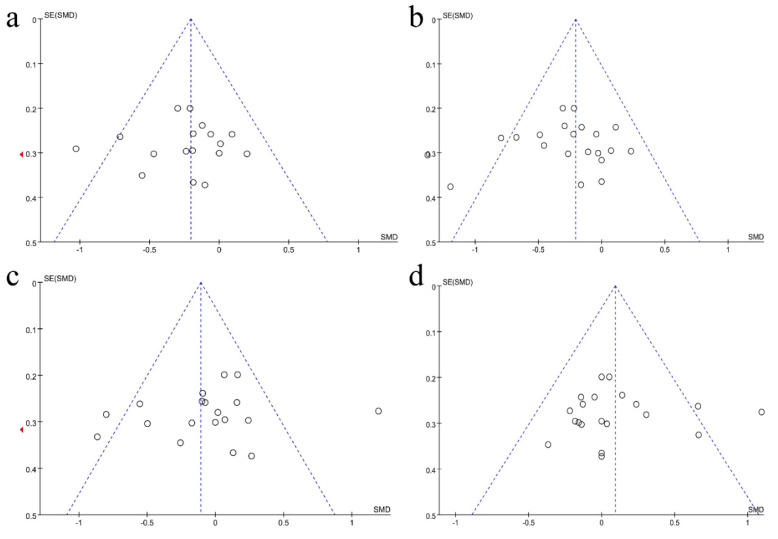
Funnel plots demonstrating publication bias in the included RCTs reporting the effects of probiotic intake on dyslipidemia. Note: Each dot represents a different study. (**a**) TC levels; (**b**) TG levels; (**c**) LDL-C levels; (**d**) HDL-C levels.

As shown by the funnel plots in Figure 7, the effect sizes were symmetrically distributed around the pooled effect size for TC (Figure 7a) and TG (Figure 7b), but not for LDL-C (Figure 7c) and HDL-C (Figure 7d). Thus, we have performed the Egger’s regression intercept test for detecting the potential publication bias. The results showed that the *p* values of LDL-C and HDL-C are 0.345 and 0.711, respectively, suggesting there is no significant publication bias in the study.

### 3.6. Adverse Events

In this meta-analysis, four studies reported adverse events, including minor gastrointestinal disturbances [37], gastrointestinal symptoms, infection, hypoglycemia, headache and musculoskeletal symptoms [39], flatulence [19] and abdominal discomfort [41], due to probiotics. One study reported no adverse effects of probiotic supplementation, but three participants in their study reported higher sexual desire after the study [20]. In two trials, the T2DM patient compliance to the probiotic treatments was good, and no serious adverse event was reported [32,33].

## 4. Discussion

Dyslipidemia, a large range of lipid abnormalities, may involve a combination of increased TC, LDL-C and TG levels, or decreased HDL-C level [42]. It is considered as a main risk factor for the occurrence and development of cardiovascular disease (CVD) in T2DM patients [42]. Notably, insulin resistance is a main factor for atherosclerotic CVD, cerebrovascular accident, and peripheral arterial disease [43], which could increase concentrations of plasma TG and LDL-C and reduce concentrations of HDL-C [44]. Previous studies have shown that the severe impairment of HDL function could further increase the risk of CVD [45]. In addition, the nature of LDL particles in patients with diabetes is more atherogenic than in those patients who are nondiabetic [46]. Based on these results, some studies considered that the reduction in HDL-C [45] and increase in LDL-C [47] indicate an increasing risk of CVD. Thus, regulating plasma lipid concentrations could be helpful in alleviating T2DM. Recently, increasing attention has been paid to probiotics due to their potential role in alleviating dyslipidemia in T2DM patients. Some studies have demonstrated that probiotics intake could inhibit the host absorption of dietary cholesterol and suppress the reabsorption of bile acid in the small intestine [48]. Probiotics may help break down food-derived indigestible carbohydrates and increase the production of short-chain fatty acids (SCFAs) [49]. The resultant SCFAs could contribute to decreasing the cholesterol concentrations, either by inhibiting hepatic cholesterol synthesis or redistributing cholesterol from plasma to the liver [49].

In this meta-analysis, 2 [30,33] of the included studies were omitted because of high heterogeneity, leaving 13 eligible studies for analysis. The results demonstrated that probiotic intake could reduce TG and TC concentrations. This finding is in line with those of another one meta-analysis [50]. They included 11 RCTs in their study, 7 of which were also included in our meta-analysis. These similar results confirm the role of probiotics in reducing TC and TG concentrations in T2DM patients. A published meta-analysis by Li et al. [17] demonstrated that probiotic intake could significantly increase HDL-C levels, but showed no significant effect on the TC, TG and LDL-C concentrations. Our study showed that the effects of probiotics intake on LDL-C and HDL-C concentrations were not statistically significant. High risks of bias were found in most of the RCTs included in the study by Li et al. [17], along with high heterogeneity between the individual analyses, which may explain this discrepancy. Notably, 2 of the 12 studies from the meta-analysis by Li et al. [17], which were of high-quality, and two recently published RCTs were also included in this study.

The results of the subgroup analysis based on the type of probiotic intervention revealed that probiotic intake in powder form, but not liquid form, could significantly reduce TC and TG concentrations. A similar result was reported by Ivey et al. [51], who found that probiotic intake via capsules, but not via yoghurt, could significantly increase the fasting glucose concentration. These results support the finding that the powder form could be a better choice for probiotic supplementation. However, as the composition of probiotic products is very complex, the benefits may be attributable to ingredients other than the probiotics themselves. Therefore, more trials with specified ingredients of probiotic products are needed to verify the exact roles of those ingredients.

Several previous studies have demonstrated that Lactobacillus plantarum PH04 intake reduces TC (7%) and TG (10%) concentrations [52]. *Enterococcus faecium* CRL 183 and *L. helveticus* 416 consumption were found to reduce TC, non-HDL-C (LDL + IDL + VLDL cholesterol fractions) and electronegative LDL levels [53]. This phenomenon may be attributable to the deconjugation of bile via a complex process, which includes bile salt hydrolysis, the binding of cholesterol to cellular surfaces and the coprecipitation of cholesterol with deconjugated bile [54]. These studies confirmed the role of probiotics in regulating blood lipid profiles, but also suggested that this role varies depending on the probiotic strains used. In our study, multispecies probiotic supplementation was found to be more effective than single-species probiotic treatment. A similar result was found in the meta-analysis by Hu et al. [50], which demonstrated that multiple species of probiotics and longer interventions (≥8 weeks) had a greater beneficial impact in terms of alleviating lipid profiles. The superiority of multispecies probiotics may result from synergistic interactions between individual species with different therapeutic activities [55].

A series of studies have demonstrated that probiotic consumption may decrease the LDL-C concentrations [16,56] and increase HDL-C concentrations [17]. However, our meta-analysis demonstrated that probiotic intake was not beneficial in regulating the LDL-C and HDL-C concentrations. This contradictory conclusion may result from the variations in experimental design and participant characteristics between the included studies. All of the results from our meta-analysis indicate that more well-designed RCTs are needed to conclusively determine which probiotic strains are more effective in alleviating dyslipidemia in T2DM.

This meta-analysis has some strengths. To date, there are some published meta-analysis studies on the topic of probiotics supplementation against T2DM. Some of these studies are focused on symptoms indicators such as HbA1c (Glycated hemoglobin A1c) [17], HOMA-IR (homeostasis model assessment of insulin resistance) [50], FBG (fasting blood glucose) [50,57] and FBS (fasting blood sugar) [58] et al. Compared to these studies, our manuscript pays more attention to the effects of probiotics against dyslipidemia in T2DM. The parameters including TC, TG, LDL-C and HDL-C were specifically analyzed in our study. Five of the published meta-analyses discussed indicators of dyslipidemia [17,18,50,59,60], but all of these were published before 2017. In our study, we have searched for relevant RCTs up to 2020 to provide an update analysis of the effects of probiotic supplementation against dyslipidemia in T2DM. Interestingly, we noticed that there are two recent RCTs [19,20] that have not been included in all the previous meta-analysis studies. These two studies revealed that multi-strain probiotic supplementation could significantly decrease HDL-C levels compared with baseline, which conflicts with the results of previous meta-analyses [17,50,59,60]. Therefore, we think these two references provide updated information in our present meta-analysis.

This meta-analysis has some limitations. Firstly, the funnel plots of TC, TG, LDL-C and HDL-C concentrations were not completely symmetrical, suggesting a risk of publication bias. There were variations in both experimental design and statistical methods between the included studies, which possibly led to selection bias. Furthermore, few studies performed microbiological experiments to test the viability of the probiotic species. Only four studies [37,38,39,40] performed fecal analyses to quantify the changes in intestinal microbial composition before and after the supplementation. Lastly, the observation period of some of the included RCTs was short, which was insufficient to evaluate changes in dyslipidemia in T2DM patients.

In summary, probiotic supplementation could be helpful in regulating blood lipid profiles, particularly with respect to reducing TC and TG concentrations. However, it is likely not helpful in regulating LDL-C and HDL-C concentrations. This result suggests that probiotics could be a non-pharmacological alternative for the treatment of T2DM. However, more RCTs with a larger sample size, longer research periods and a rigorous experimental design are needed in the future. Furthermore, more indicators should be included in RCTs to develop clinical practice guidelines.

## Figures and Tables

**Table 1:** Characteristics of included randomized controlled trials (RCTs) in this meta-analysis.

Author/Date	Age	No. of Participants (Intervention/Placebo)	Administered Probiotics	Whether the Included RCTs Have Measured Dyslipidemia Indicators	Type of Study	Trial Number	Country
Asemi et al., 2013 [29]	35–70	60 (27/27)	Capsules: *L. casei*, 7 × 10^9^ CFU; *L. acidophilus*, 2 × 10^9^ CFU; *L. rhamnosus*, 1.5 × 10^9^ CFU; *B. breve*, 2 × 10^10^ CFU; *B. longum*, 7 × 10^9^ CFU; *L. bulgaricus*, 2 × 10^8^ CFU; *S. thermophilus*, 1.5 × 10^9^ CFU (8 weeks).	Yes	RCT	-	Iran
Asemi et al., 2014 [30]	35–70	62 (31/31)	Synbiotic food: *L. sporogenes,* 1 × 10^7^ CFU (each day for 6 weeks).	Yes	RCT	IRCT201201195623N1	Iran
Asemi et al., 2016 [31]	35–70	51 (25/26)	Synbiotic food: 1 × 10^7^ CFU, *L. sporogenes* (three times a day for 6 weeks).	Yes	RCT		Iran
Ebrahimi et al., 2017 [32]	35–75	70 (35/35)	Capsules: *Lactobacillus*; *Bifidobacterium* family, *S. thermophilus* (500 mg/d for 9 weeks).	Yes	RCT	IRCT2015072223284N1	Iran
Ejtahed et al., 2011 [33]	30–60	60 (30/30)	Yogurt: *L. acidophilus* La5; *B. lactis* Bb12 (300 g/day for 6 weeks).	Yes	RCT	IRCT 138903223 533N1	Iran
Feizollahzadeh et al., 2016 [34]	35–68	40 (20/20)	Soy milk: *L. plantarum* A7, 2 × 10^7^ CFU (200 mL milk/day for 8 weeks).	Yes (lack of TC level)	RCT	IRCT201405265062N8	Iran
Firouzi et al., 2017 [37]	30–70	101 (48/53)	Sachet: *L. acidophilus,* 3 × 10^10^; *L. lactis*, 3 × 10^10^; *B. bifidum*,3 × 10^10^; *B. longum,* 3 × 10^10^; *L. casei,* 3 × 10^10^; *B. infantis,* 3 × 10^10^ (10^10^ cfus/day for 12 weeks).	Yes	RCT	NCT01752 803	Malaysia
Hsieh et al., 2018 [38]	25–70	68 (46/22)	Capsules: *L. reuteri* ADR-1 or *L. reuteir* ADR-3, 2 × 10^9^ CFU or 1 × 10^10^ cells (9 months).	Yes	RCT	NCT02274272	China
Mazloom et al., 2013 [35]	25–65	34 (16/18)	Capsules: *L. bulgaricus*, *L. bifidum*, *L. acidophilus*, *L. casei* (3000 mg/day for 6 weeks).	Yes	RCT	-	Iran
Mobini et al., 2017 [39]	50–75	44 (15/high dose 14/low dose 15)	Probiotic powder: *L. reuteri* DSM 17938, low (10^8^ CFU/day) or high dose (10^10^ CFU/day) (one dose per day for 12 weeks).	Yes	RCT	NCT01836796	Sweden
Ostadrahimi et al. 2015 [36]	35–65	60 (30/30)	Fermented milk: *L. casei*, *L. acidophilus* and *Bifidobacteria* (600 mL/day for 8 weeks).	Yes	RCT	IRCT2013 07092017N 14	Iran
Razmpoosh et al., 2019 [20]	30–75	60 (30/30)	Capsule: *L. acidophilus*, 2 × 10^9^ CFU; *L. rhamnosus*, 1.5 × 10^9^ CFU; *L. casei* 7 × 10^9^ CFU; *L. bulgaricus*, 2 × 10^8^ CFU; *B. breve*, 3 × 10^10^ CFU; *B. longum*, 7 × 10^9^ CFU; *S. thermophilus*, 1.5 × 10^9^ CFU (6 weeks).	Yes	RCT	IRCT2013100714925N1	Iran
Sabico et al., 2019 [19]	30–60	61 (31/30)	Freeze-dried powder: *B. lactis* W52, 2.5 × 10^9^ cfu/g; *L. acidophilus* W37, 2.5 × 10^9^ cfu/g; *L. brevis* W63, 2.5 × 10^9^ cfu/g; *B. bifidum* W23, 2.5 × 10^9^ cfu/g; *L. casei* W56, 2.5 × 10^9^ cfu/g; *L. salivarius* W24, 2.5 × 10^9^ cfu/g; *L. lactis* W58, 2.5 × 10^9^ cfu/g; *L. lactis* W19 2.5 × 10^9^ cfu/g (twice daily for 6 months).	Yes	RCT	NCT01765517	Saudi Arabia
Sato et al., 2017 [40]	30–79	68 (34/34)	Milk: *L. casei*, 4 × 10^10^(80-mL bottle milk fermented with one bottle of milk every day for 16 weeks).	Yes (lack of TC and LDL-C levels)	RCT	UMIN000018246	Japan
Tonucci et al., 2015 [41]	35–60	45 (23/22)	Fermented milk: *B. lactis* BB12 and *L. acidophilus* LA5 (6 weeks).	Yes	RCT	Ensaiosclini cos.gov.br/ rg/RBR-219644	Brazil

Note: S, Streptococus; B, Bifidobacterium; L, Lactobacillu.

**Table 2:** Results of subgroup analyses for TC, TG, LDL-C and HDL-C levels in T2DM patients.

Subgroup	TC				TG				LDL				HDL			
	No	Pooled Effect (95% CI) mmol/L	I^2^	*p*	No	Pooled Effect (95% CI) mmol/L	I^2^	*p*	No	Pooled Effect (95% CI) mmol/L	I^2^	*p*	No	Pooled Effect (95% CI) mmol/L	I^2^	*p*
Overall analysis	17	−0.23 (−0.37, −0.10)	11	0.0009	20	−0.27 (−0.44, −0.11)	45	0.001	18	−0.11 (−0.26, 0.04)	26	0.14	20	0.09 (−0.06, 0.24)	36	0.22
Number of strains																
<2	7	−0.07 (−0.30, 0.16)	0	0.58	10	−0.06 (−0.24, 0.12)	0	0.50	8	−0.04 (−0.28, 0.20)	16	0.75	10	0.03 (−0.15, 0.21)	0	0.73
≥2	10	−0.32 (−0.52, −0.12)	34	0.001	10	−0.46 (−0.71, −0.21)	58	0.0003	10	−0.16 (−0.36, 0.04)	36	0.11	10	0.14 (−0.11, 0.39)	60	0.28
Duration (week)																
<8	5	−0.18 (−0.41, 0.05)	0	0.13	5	−0.37 (−0.68, −0.07)	38	0.02	5	−0.06 (−0.29, 0.17)	0	0.61	5	0.04 (−0.20, 0.27)	0	0.77
≥8	12	−0.25 (−0.43, −0.07)	23	0.006	15	−0.24 (−0.43, −0.04)	49	0.02	13	−0.13 (−0.32, 0.07)	37	0.20	15	0.12 (−0.07, 0.31)	46	0.23
BMI																
<29	9	−0.12 (−0.31, 0.06)	0	0.19	12	−0.13 (−0.29, 0.04)	9	0.14	10	−0.12 (−0.31, 0.07)	10	0.21	12	−0.01 (−0.16, 0.15)	0	0.94
≥29	8	−0.34 (−0.57, −0.11)	33	0.003	8	−0.48 (−0.75, −0.20)	53	0.0006	8	−0.10 (−0.36, 0.15)	46	0.41	8	0.24 (−0.06, 0.54)	61	0.12
Type of intervention																
Liquid	2	−0.23 (−0.63, 0.16)	5	0.25	5	−0.04 (−0.27, 0.20)	0	0.76	3	−0.37 (−0.97, 0.23)	69	0.22	5	−0.00 (−0.27, 0.26)	21	0.99
Powder	15	−0.23 (−0.39, −0.08)	18	0.003	15	−0.36 (−0.56, −0.15)	51	0.0005	15	−0.07 (−0.21, 0.07)	5	0.34	15	0.12 (−0.06, 0.30)	40	0.18

Abbreviations: CI, confidential interval; No., number of included studies; *p*, value for heterogeneity within subgroup; I^2^, heterogeneity.

## Supplementary Materials

The following are available online at https://www.mdpi.com/2304-8158/9/11/1540/s1, Table S1: The Systematic Reviews and Meta-Analyses (PRISMA) statement of the present meta-analysis.
